# The Effect of Big Data-Based Digital Payments on Household Healthcare Expenditure

**DOI:** 10.3389/fpubh.2022.922574

**Published:** 2022-06-02

**Authors:** Chengming Li, Daming Li, Si He, Shiqi Sun, Yuan Tian, Zeyu Wang

**Affiliations:** ^1^School of Economics, Minzu University of China, Beijing, China; ^2^School of Software & Microelectronics, Peking University, Beijing, China; ^3^Center for Enterprise Growth and National Economic Security Research, Tsinghua University, Beijing, China; ^4^School of Public Administration, Guangzhou University, Guangzhou, China

**Keywords:** big data, digital payments, household healthcare expenditure, credit constraints, social capital

## Abstract

Whether families using big data-based digital payments will increase household healthcare expenditure is a subject that needs to be investigated in the era of big data. Based on the data from China Family Panel Studies (CFPS), 24,126 samples from 2014 to 2018 are used to examine the impact and mechanism of big data-based digital payments on household healthcare expenditure. The empirical results of this paper show that the use of digital payments by households can significantly increase household healthcare expenditure with the empowerment of big data. This research employs the instrumental variable method to verify and produce consistent estimation results in order to address potential endogeneity issues such as measurement error and missing variables. We learn via mechanism analysis that household adoption of big data-driven digital payments can remove credit limitations and build social capital, resulting in higher household health-care spending. We also perform a heterogeneity analysis. The findings reveal that when a family's traditional financial accessibility is high, the head of the household is young or middle-aged, and the head of the household has a higher level of education, digital payment will play a larger role in encouraging household healthcare expenditure. The conclusions of this paper are still solid after changing the indicators of household healthcare expenditure substituting the indicators of digital payment, and adjusting the variables. As a result, this article provides micro-evidence for the usage of digital payments by households to enhance healthcare spending.

**JEL Classification:**

D12 G21 O30 O53 I12

## Introduction

Household healthcare expenditure have risen to become the third greatest family expense after housing and education, and the phenomenon has been attracting the attention of many academics. Economic strife (1), family income (2–6), age and gender (7, 8), microfinance (9, 10), and other factors have been identified to influence household healthcare expenditure in previous studies.

Health problems are growing more serious as China's economy grows, and households are paying greater attention to medical treatment. The share of families working overtime and staying up late in Beijing is substantially higher than the general average level, according to the survey results of 2021 Chinese Urban Family Health Life Power White Paper. Families in Beijing and Shanghai are burdened by a greater economic load, while families in Zhengzhou are plagued by mental illness and financial strain. Families are increasingly confronted with acute mental sub-health and intense pressure in an era when individuals are more concerned about their health. Health security is becoming an increasingly significant aspect of family health management. For example, 65 percent of families agree that customizing commercial health insurance is vital. When compared to traditional payment methods, digital payment based on big data is more favorable to breaking down time and space barriers and lowering the threshold for consumers to enjoy financial services, allowing them to do so more conveniently and swiftly. Digital medicine has become an active choice to alleviate the shortage of medical resources and limited medical conditions, thanks to the empowerment of digital technologies like big data, cloud computing, and blockchain. Digital payment has a bigger role to play in the growth of digital healthcare. As a result, from the standpoint of big data, this research investigates the effect of big data-based digital payments on household healthcare expenditure.

On the one hand, households' use of digital payment based on big data can broaden available financial services, increase sources of income, and alleviate credit constraints; on the other hand, we can accumulate social capital, broaden information channels, and reduce transaction costs, which may lead to an increase in household healthcare expenditure. However, existing research does not provide a clear response to the question of how digital payments based on big data affect household health care spending. The majority of studies look at the impact of family income (6), age and gender (8), and microfinance (9) on family healthcare expenditure, or address the potential benefits and risks of digital payment (11) and the impact of digital payment on rural household consumption in China (12, 13). Of course, some articles focus specifically on the impact of mobile money on household medical spending (14). However, there are few papers that look at the influence of digital payments on household health care spending from a big data viewpoint.

As a result, utilizing the China Family Panel Studies (CFPS) database and 24,126 family sample data from 2014 to 2018, this research investigates the influence of digital payment on household healthcare expenditure from a big data viewpoint, yielding the following findings. First, this research shows that digital payment enabled by big data can boost household health care expenditures using OLS estimation. Second, we use the instrumental variable technique to verify and get consistent estimation results in order to address potential endogenous issues such as measurement errors and missing data. Third, the mechanism analysis reveals that digital payments enabled by household big data applications can reduce credit limitations and build social capital, resulting in an increase in household health-care spending. Fourth, the heterogeneity analysis reveals that the use of digital payment based on big data is more effective in encouraging household healthcare expenditure in families headed by middle-aged and young individuals with a high level of education and financial accessibility. The conclusions of this research are still solid after replacing the indicators of household healthcare expenditure, replacing the indicators of digital payment, and tail reduction. As a result, this article presents micro evidence encouraging families to adopt digital payment to boost their household healthcare expenditure.

The following are the contributions of this paper. First, this paper adds to the body of knowledge about the factors that influence household healthcare expenditure (5, 6, 8, 9). The existing literature focuses mostly on home consumption studies and does not consider the influence of household health care spending. Song et al. (15) and Li et al. (16), for example, looked at the relationship between digital finance and household consumption from a digital finance perspective; Hou et al. (17) believed that liquidity constraints would affect household consumption; Kang (18) believed that social networks would increase household consumption. Furthermore, a few studies have looked into the characteristics that influence household healthcare expenditure. Microfinance (9), family income (5, 6), and age and gender (8) all have an impact on household healthcare expenditure. This study investigates the influence of digital payment based on big data on household healthcare expenditure and clarifies the impact of payment mode shift on household healthcare expenditure in the digital era through empirical analysis.

Second, this research adds to the body of knowledge about the influence of digital payments on household spending. The majority of existing work focuses on the possible benefits and risks of digital payment (11), the influence of digital payment on Chinese households' overall spending (12, 13), etc. There are, of course, certain studies that look specifically at the influence of mobile money on household health-care spending (14). However, there is limited literature on the influence of digital payment on household healthcare expenditure under the empowerment of big data from a big data perspective. As a result, this work adds to the body of knowledge about the influence of digital payments on household consumption, as well as offering new perspectives and research ideas in this area.

Third, this study adds to the body of knowledge about the impact of Fintech on family economic behavior. The impact of consumer finance (19, 20) and technology finance (21–23) on family economic behavior is the subject of most existing studies. Digital payment is an important financial technology tool, but there is little research on its impact on family healthcare behavior. This research provides new evidence regarding the impact of Fintech on family economic behavior based on this, from the standpoint of digital payment, along with the enabling function of big data.

This paper's structure is as follows: The theoretical analysis of digital payment on household healthcare expenditure under big data empowerment is summarized in Section Theoretical Analysis and Research Hypotheses; Section Data and Methods provides the data, variables, and models used in empirical analysis; Section Results presents the major result, which includes a mechanism and robustness analysis; Section Further Discussion: The Heterogeneity of Household Healthcare Expenditures presents the paper's further analysis; and Section Conclusion presents the conclusion.

## Theoretical Analysis and Research Hypotheses

### Household Use Big Data-Based Digital Payments Can Ease Credit Constraints and Thus Increase Household Healthcare Expenditure

Financial institutions gain access to a large amount of household data after households use big data-based digital payments. On the one hand, financial institutions can use big data from households to understand households' consumption preferences and accurately deliver personalized information by mining households' consumption data in order to increase households' demand for financial services (24); on the other hand, financial institutions can use big data analytics (BDA) to build decentralized and distributed models to predict households' credit default probabilities (25). As a result, big data-driven digital payments can affect both the supply and demand sides of financial services to ease household credit constraints encouraging households to use healthcare services (26).

On the demand side of financial services, households' use of big data-based digital payments helps them increase their financial and entrepreneurial incomes (12), while higher incomes increase the likelihood of successful credit access and help alleviate credit constraints. On the one hand, financial institutions use big data to push appropriate financial products to households based on their characteristics and needs in order to increase household financial income. Households can better manage their finances with the help of big data-based digital payments, such as purchasing money funds and investing in equity funds, thereby increasing their financial income. On the other hand, big data based-digital payments can stimulate households' entrepreneurial enthusiasm and thus increase their entrepreneurial income by providing functions such as information transfer, credit, and social interaction (27, 28).

On the supply side of financial services, on the one hand, financial institutions analyze households' credit, consumption, social, and other behaviors based on big data generated by digital payments (29) to accurately grasp households' credit status (30, 31) and include creditworthy but without collateral households into the scope of credit allocation and expand the coverage of financial service supply. Financial institutions, on the other hand, can use big data to better control the risk of credit disbursement, lower the cost of credit risk, and exit more low-threshold credit services. As a result, households can use microfinance services provided by digital platforms like WeChat and Alipay to gain access to more flexible, convenient, and low-threshold credit, easing their credit constraints (32). Traditional payment tools can achieve microfinance mobilization in the short term, but some households will face problems such as financial exclusion due to high barriers erected by financial institutions and a lack of awareness among some rural households, which will not be able to alleviate credit constraints (33–35).

In conclusion, households' use of big data-based digital payments can boost household healthcare expenditure by increasing income sources from both the demand and supply sides. As a result, this paper proposes hypothesis below.

**H1: Households use big data-based digital payments to alleviate credit constraints and thus boost household healthcare expenditure**.

### Household Use Digital Payments Based on Big Data Can Increase Social Capital and Thus Increase Household Healthcare Expenditure

Digital platforms in China, such as WeChat and Alipay, offer not only digital payment services but also virtual social features. When households use big data-based digital payments, these digital platforms tend to use big data, based on a MapReduce-based paradigm, to perform social data analysis through the most popular framework, Apache Hadoop, to drive the creation and strengthening of social networks among different households on digital platforms to increase the viscosity of households' use of digital platforms to increase the viscosity of households' use of digital platforms to increase the viscosity of households' use of digital platforms (36). The formation and strengthening of social networks increase not only the viscosity of families' use of digital platforms, but also their social capital. In other words, households' use of big data-based digital payments on increases the frequency of communication with friends, relatives, and other subjects, thereby expanding and strengthening household social networks and contributing to the accumulation of social capital (37). As a result, increasing household social capital facilitates the expansion of information channels and the reduction of transaction costs, which increases household healthcare expenditure.

Households' use of big data-based digital payments expands access to information. Households are connected to digital platforms and their social capital grows when they use big data-based digital payments. Following that, digital platforms like WeChat and Alipay can push more healthcare-related information to households, increasing their access to information. Households can purchase products such as health insurance and health consultation based on information pushed to the Internet by big data. Furthermore, digital payments make it easier for households to buy healthcare-related products and services by streamlining the transaction process by allowing payments and transfers to be made via the Internet (22, 38).

Furthermore, households can reduce transaction costs in healthcare visits by using big data-based digital payments. When households build social capital through big data-based digital payments, they become more connected to subjects like family and friends, other households, and hospitals, giving them more opportunities and resources while lowering transaction costs (19, 39). Hospitals can use big data from households to analyze the health needs of households in real time and provide remote registration and online medical services as needed, establishing triage mechanisms and personal medical databases for patients and adjusting resource allocation, making it more efficient and convenient for patients to see a doctor and, to some extent, reducing the transaction costs of household healthcare (40). It is also easy for banks to assess the creditworthiness of potential borrowers using big data from households, allowing them to provide more accurate and cheaper credit to households (41), lowering the transaction costs of credit between banks and households.

In conclusion, households' use of big data-based digital payments can reduce widening information channels, lower transaction costs, increase social capital, and thus boost household healthcare expenditure. Thus, this paper proposes hypothesis below.

**H2: households' use of big data-based digital payments can accumulate social capital, boosting household healthcare expenditure**.

### Different Types of Household Applications of Big Data-Based Digital Payments Have Heterogeneous Impact on Household Health Care Expenditure

Furthermore, the use of big data-based digital payments by households has a heterogeneous effect on household health care expenditures, according to our study. Financial literacy is influenced by socio-demographic characteristics (e.g., age, education) in households (42), and because big data-based digital payments are a key component of digital finance, financial literacy influences households' use of big data-based digital payments. In terms of age differences, young and middle-aged people typically have higher cognitive levels, and higher cognitive levels significantly increase wage income (43, 44). Increased income allows this group to use big data-based digital payments more frequently, which boosts household income (26). Due to physical aging, the cognitive level of household heads declines as they grow older. As a result, young and middle-aged heads of households typically have a higher level of cognition than older heads of households, and are able to use and enjoy the benefits of big data-based digital payments more quickly, resulting in higher household healthcare expenditure. When it comes to education, the more educated group typically has better learning abilities and a larger knowledge base (45). Big data-based digital payments are novel and distinct from traditional payments, and they operate at a certain threshold, requiring users' ability to learn. Without assistance, the less educated may not be able to use or comprehend the features of big data-based digital payments, and may be hesitant to use them. As a result, higher education lowers the barrier to household heads using big data-based digital payments (46).

The use of big data-based digital payments is more prevalent in promoting household health care expenditures among households living in areas with greater financial accessibility on a regional level. Financial exclusion has long been a problem in rural areas of the country with distinct geographic characteristics (47), and the presence of financial institution outlets can effectively mitigate financial exclusion (47, 48). In other words, if households live in areas with limited access to traditional financial services, they are more likely to be financially excluded due to their remote location. Households can enjoy traditional financial services more conveniently and have good habits of participating in financial services in places with convenient transportation and high accessibility to traditional financial services, making them more likely to accept big data-based digital payments and play the role of big data-based digital payments in promoting household healthcare expenditure.

**H3: The use of digital payments contributes more to household healthcare expenditure if the household has higher traditional financial accessibility, the household head is young or middle-aged, and the household head is more educated**.

## Data and Methods

### Model Setting

Referring to Liu et al. (49) and Zhang et al. (13), this paper develops an empirical model to explore the impact of digital payment use on household healthcare expenditure. The specific model is set up as follows.


(1) $$\begin{array}{l} Expenditure_{ijt}=\alpha_{0}+\beta_{0}Digital\;payments_{ijt}+\gamma_{0}Control_{ijt}\\ \;+\varphi_{i}+\delta_{t}+\varepsilon_{ijt} \end{array}$$

The explanatory variable *Expenditure*_*ijt*_ is household healthcare expenditure from the *t* th year and *j* th province and *i* th household. The household healthcare expenditure in the past year is obtained by logarithmic treatment. The core explanatory variable *Digital payments*_*ijt*_ is the big data-based digital payment, which is represented by the frequency of Internet business activity of the household head in the usual case. We focus mainly on the coefficient β_0_ to see the impact of digital payments on household healthcare expenditure. A positive coefficient indicates that big data-based digital payments boost household healthcare expenditure. If the coefficient is negative, it indicates that digital payments reduce household healthcare expenditure. *Control*_*ijt*_ is the household head-level and household-level control variables, φ_*i*_ denote household fixed effects, and δ_*t*_ denote time fixed effects. Since households may be correlated in the same province, we cluster the standard errors to the provincial level.

### Indicator Metrics

#### Explanatory Variable: Household Healthcare Expenditure

The explanatory variable is household healthcare expenditure. Healthcare expenditures are divided into two categories in the CFPS data: household medical expenditure and household fitness expenditure. Specifically, the CFPS questionnaire addresses household healthcare costs by asking, “What medical expenditure were paid directly by your household in the last 12 months? Of these, fitness expenditure do not include expenses that have been repaid or are expected to be reimbursed, but they do include certain expenses borrowed from friends and relatives or previously paid.” “How much did your family pay for healthcare in the past 12 months? Healthcare expenditures include, among other things, the cost of physical activity and the purchase of related equipment and healthcare items.” It can be observed that the first question indicates the household's medical care spending, while the second question reflects the household's healthcare spending, which combined show the significance of healthcare to the household. To get the logarithmized result, we add up the medical and healthcare costs paid directly by households in the previous 12 months to get a total, then add 1 to the total and take the natural logarithm of the value after adding 1 to get the result.

#### Core Explanatory Variables: Digital Payments

The frequency of our household head's Internet business activity is used as a proxy variable for the extent to which the household uses digital payments. The household head is the primary decision-maker in the household, so the behavior of the household head has a significant impact on household activities. Specifically, this variable is addressed in the CFPS questionnaire by the question, “In general, how often do you use the Internet network for business activities (e.g., using Internet banking, online shopping)?” To provide a more visual representation of the extent to which households use digital payments, we assign a value to the frequency of the household head's response. We use 6 if the home head utilizes the Internet virtually every day for work purposes. We use 5 if the household head uses it 3 to 4 times a week; 4 if the household head uses it 1–2 times a week; 3 if the household head uses it 2–3 times a month; 2 if the household head uses it once a month; 1 if the household head uses it once every few months; and 0 if the household head never uses digital payments.

#### Other Control Variables

In terms of the selection of control variables, this paper draws on existing literature (12, 13, 23, 50–52) to control for both household-level influences and relevant variables at the household head- level. Specifically, at the household level, we control for household size, child support ratio, elderly support ratio, and net household income; at the household head level, this paper controls for the age of the household head and its squared term, the gender of the household head, the marital status of the household head, and whether the household head has party membership.

Specifically, the larger the household, the higher the household healthcare expenditure (53); the larger the proportion of children and the proportion of elderly, the higher the household's dependency burden, which may also contribute to household healthcare expenditure (54); the higher the household's net income, the higher the household's healthcare (6); and the age, gender, marital status, and party membership of the household head may also have a significant impact on household healthcare expenditures (8, 55).

### Data Sources and Descriptive Statistical Analysis

The data we utilize comes from the China Family Panel Studies (CFPS) database, which is updated every 2 years. Because the most recent released household data from this survey are available up to 2018, and digital payments have been widely utilized since 2013, we selected to use data from 2014 to 2018. Referring to existing research methods (56), we processed the data as follows: (1) We eliminated samples with key codes missing, such as household, person, urban-rural, and provincial codes. (2) Since we used panel data and the distribution of data within each household is not necessarily the same, these data may show linearly varying relationships over the survey period. As a result, we used linear interpolation to fill in certain blanks (e.g., net household income per capita and age). Variables that were missing for three consecutive periods and could not be filled in were also eliminated. In addition, samples with incomplete gender and household size are excluded. This is because the household head's gender does not change, and changing the family size is difficult in the short term, therefore the interpolation technique is not applicable. In addition, we logarithmized variables such household expenditures and household net income (adding 1 to the values of the variables and taking the natural logarithm of the new values obtained after the addition of 1 to obtain the logarithmized results). (3) Since the CFPS does not give explicit information on the household head, researchers usually treat the principal, decision-maker, property owner, and financial respondent as the household head. However, since 2014, as information such as principal is no longer disclosed in the CFPS, only the financial respondent and the property owner in the household can be the head of the household, and most studies treat the financial respondent as the head of the household. Therefore, this paper identifies household heads by financial respondents in 2014 and only retains information on household heads who are 16 years old or older. (4) We logarithmized variables with large values such as income and distance. Specifically, we add 1 to their values and then take the natural logarithm to obtain the new variables. (5) For continuity in the time dimension, we only keep households that were surveyed in all 3 years. In addition, the nature of a household may fundamentally change when a household is split into multiple households or when a household moves, so in this paper we only retain households that were not split and did not move during the sample period.

After the above processing, we obtained 24,126 samples between 2014 and 2018, and the descriptive statistical results of the relevant variables are shown in Table 1. Among the household head and household control variables, the mean value of age of household head is 52.98, the standard deviation is 13.15, the minimum value is 16, and the maximum value is 93; the mean value of household size is 3.786, the standard deviation is 1.876, the minimum value is 1, and the maximum value is 21; the mean value of proportion of children is 0.124, the standard deviation is 0.201, the minimum value is 0, and the maximum value is 4; the mean value of elderly dependency ratio is 0.24. The above data indicate that the age distribution of household heads is wide, but the household structure is relatively similar.

**Table 1:** Statistical description of variables.

**Variable**	**Explanation**	***N***	**Mean**	**Sd**	**Min**	**Max**
Healthcare expenditure	The sum of household medical expenditure and household health expenditure	24,126	6.901	2.711	0	13.84
Digital payments	Extent of digital payments used by the household head	24,126	0.460	1.276	0	6
Age of the household head	Age of the household head	24,126	52.98	13.15	16	93
Age^2^ of the household head	Age^2^/100 of the household head	24,126	29.80	14.13	2.560	86.49
Gender of the household head (male = 1)	Gender of the household head	24,126	0.514	0.500	0	1
Marriage of the household head (married = 1)	Marital status of the household head	24,126	0.870	0.336	0	1
Party membership (party = 1)	Whether the household head is a party member	24,126	0.107	0.309	0	1
Household size	Number of families eating together	24,126	3.786	1.876	1	21
Child ratio	Number of child in the household size	24,126	0.124	0.201	0	4
Elderly ratio	Number of elderly in the household size	24,126	0.240	0.350	0	3
Net household income	Net income is the income after deducting the cost of agricultural production	24,126	9.007	1.576	0	13.83

*We define a child as an individual who is not older than 16 years old, an elderly person as an individual who is 60 years old and older*.

## Results

### Baseline Regression

We present the results of estimating the impact of digital payments on household healthcare expenditure in Table 2. Specifically, column (1) shows the regression results without any control variables, column (2) shows the regression results after controlling for household and time fixed effects, column (3) shows the regression results after adding household head-related variables to column (2), and column (4) shows the regression results after further adding household-related variables. As seen in column (1), the regression coefficient of digital payment is 0.0844, which is significant at the 1% statistical level. As seen in columns (2), (3), and (4), this paper still shows a significant positive effect of digital payments based on big data on household healthcare expenditure after controlling for household and time fixed effects as well as the household head and household level influences.

**Table 2:** Baseline regression results.

	**(1)**	**(2)**	**(3)**	**(4)**
Digital payments	0.0844***	0.0591**	0.0594**	0.0534**
	(5.90)	(2.36)	(2.19)	(2.07)
Age of the household head			−0.0170	−0.0410
			(−0.28)	(−0.61)
Square of age of the household head/100			0.0175	0.0387
			(0.36)	(0.79)
Gender of the household head			0.981	0.855
			(1.54)	(1.51)
Marriage of the household head			0.685***	0.554***
			(3.78)	(3.01)
Party membership			−0.0226	−0.0416
			(−0.11)	(−0.22)
Household size				0.224***
				(9.98)
Child ratio				−0.317
				(−1.55)
Elderly ratio				−0.0492
				(−0.34)
Net household income				0.0642***
				(3.32)
Household fixed effects	No	Yes	Yes	Yes
Time fixed effects	No	Yes	Yes	Yes
*N*	24,126	24,126	24,126	24,126
*R* ^2^	0.001	0.004	0.005	0.016

^***^ and ^**^ indicate significant at the 1 and 5% statistical levels, respectively, with t-values in parentheses.

Next, we observed the coefficients of the control variables and obtained the following results. The positive coefficient on marriage indicates that married heads of households value family protection and are more willing to spend on family healthcare; the coefficient on the number of household members is also positive, indicating that the larger the family size, the higher the household healthcare expenditure; and the coefficient on net household income is also significantly positive, indicating that households with higher income also value household healthcare, and increase expenditure more.

### Robustness Analysis

#### Endogenous Processing

Due to omitted variables, this study may have endogeneity issues. In other words, variables other than those already controlled for in this paper may be associated with the use of digital payments or with households' health care expenditure, but we do not control for these other variables. In addition to this, there may be some error between the CFPS survey data and the actual household sentiment data, so there is also the possibility of measurement error in this paper.

Therefore, this paper uses the instrumental variable method to avoid the endogeneity problem. In this paper, the spherical distance from the capital city of the household's province to Hangzhou is taken as an instrumental variable. If Alipay originates in Hangzhou, the further the household is from Hangzhou, the less the use of digital payment. Therefore, we consider that the distance from the provincial capital city to Hangzhou is highly correlated with digital payment usage of households. In addition, the spherical distance is strongly exogenous and is not affected by digital payment use of households. The specific results are shown in Table 3.

**Table 3:** Robustness test: endogeneity treatment.

	**(1)**	**(2)**	**(3)**	**(4)**
	**Digital payments**	**Household healthcare expenditure**	**Digital payments**	**Household healthcare expenditure**
Digital payments		0.952^***^		1.046^***^
		(3.26)		(3.42)
Driving distance	−142.296 ^***^			
	(−3.66)			
Spherical distance			−1.145^***^	
			(−4.00)	
Control variables for household head	Yes	Yes	Yes	Yes
Control variables for household	Yes	Yes	Yes	Yes
Household fixed effects	Yes	Yes	Yes	Yes
Time fixed effects	Yes	Yes	Yes	Yes
*N*	24,126	24,126	24,126	24,126
*R* ^2^	0.130	0.083	0.130	0.105
Wald value		56.520		58.313
first stage F-value		88.11		91.55

*** *Indicate significant at the 1% statistical levels, respectively, with t-values in parentheses*.

The results show that the F-value of the weak instrumental variable test is >10 and passes the Wald test, indicating that there is no problem of weak instrumental variables and the instrumental variables we selected are valid. In addition, driving distance is an instrumental variable similar to spherical distance. Specifically, we use the driving distance from the capital city of the household's province to Hangzhou as the instrumental variable, and the results are still robust.

#### Replacing Core Explanatory Variables

Considering that the frequency of household heads using the Internet for commercial activities is not linear, in the robustness analysis, we choose to use whether household heads conduct commercial activities via the Internet as a proxy variable for digital payments in order to eliminate the effect of uneven frequency distribution. If the household head usually does not ever conduct business activities on the Internet, then we consider that the household head has not used digital payments and the core explanatory variable is equal to 0. Conversely, if the household head usually conducts business activities on the Internet, then we consider that the household head has used digital payments and the core explanatory variable is equal to 1. We know from Table 4 that the use of digital payments by households promotes household healthcare expenditure.

**Table 4:** Robustness test: replacement of explanatory variables.

	**(1)**	**(2)**	**(3)**	**(4)**
Digital payments (0–1)	0.265***	0.218**	0.216**	0.194**
	(6.56)	(2.68)	(2.57)	(2.49)
Control variables for household head	No	No	Yes	Yes
Control variables for household	No	No	No	Yes
Household fixed effects	No	Yes	Yes	Yes
Time fixed effects	No	Yes	Yes	Yes
*N*	24,126	24,126	24,126	24,126
*R* ^2^	0.001	0.004	0.005	0.016

^***^ and ^**^ indicate significant at the 1 and 5% statistical levels, respectively, with t-values in parentheses.

#### Replacing the Explanatory Variables

Household healthcare expenditure can be divided into two components: household medical expenditure and household fitness expenditure, and both components can represent to some extent the degree to which households value their health. To conduct robustness tests, we split the household healthcare expenditure into two sub-dimensions, medical expenditure and fitness expenditure. In addition, we also replace the explanatory variables with dummy variables based on the median and mean of household healthcare expenditure. If the household healthcare expenditure exceeds the median or the mean, the explained variable equals 1. As seen in Table 5, the results are still robust.

**Table 5:** Robustness tests: replacement of explanatory variables.

	**(1)**	**(2)**	**(3)**	**(4)**
	**Medical**	**Fitness**	**Median**	**Average**
	**expenditure**	**expenditure**	**(0–1)**	**(0–1)**
Digital payments	0.0432**	0.157***	0.00840**	0.00790**
	(2.20)	(8.71)	(2.28)	(2.63)
Control variables for household head	Yes	Yes	Yes	Yes
Control variables for household	Yes	Yes	Yes	Yes
Household fixed effects	Yes	Yes	Yes	Yes
Time fixed effects	Yes	Yes	Yes	Yes
N	24,126	24,126	24,126	24,126
*R* ^2^	0.782	0.098	0.018	0.017

^***^ and ^**^ indicate significant at the 1 and 5% statistical levels, respectively, with t-values in parentheses.

#### Replacing the Sample Range

To prevent some extreme values and outliers from affecting the study, we next apply winsorizing to the sample data to correct for all variables and address possible measurement errors in the regression. We can see from the results in columns (1) to (3) in Table 6 that the use of big data-based digital payments by households increases household healthcare expenditure after a 1, 5, and 10% winsorizing process, respectively.

**Table 6:** Robustness test: replacement sample range.

	**(1)**	**(2)**	**(3)**
	**Winsorize 1%**	**Winsorize 5%**	**Winsorize 10%**
Digital payments	0.0559^**^	0.0534^**^	0.0534^**^
	(2.17)	(2.07)	(2.07)
Control variables for household head	Yes	Yes	Yes
Control variables for household	Yes	Yes	Yes
Household fixed effects	Yes	Yes	Yes
Time fixed effects	Yes	Yes	Yes
*N*	24,126	24,126	24,126
*R* ^2^	0.016	0.016	0.016

** *Indicate significant at the 5% statistical levels, respectively, with t-values in parentheses*.

### Mechanism Analysis

It can be found from the previous section that the use of big data-based digital payments by households significantly contributes to household healthcare expenditure. We further discuss and analyze the mechanism by which digital payments affect household healthcare expenditure using a mediating effects model, which is set up as follows.


(2) $$\begin{array}{l} Expenditure_{ijt}=\alpha_{1}+\beta_{1}Digital\;payments_{ijt}\\ \;+\gamma_{1}Control_{ijt}+\varphi_{i}+\delta_{t}+\varepsilon_{ijt} \end{array}$$

(3) $$\begin{array}{l} Inter_{ijt}=\alpha_{2}+\beta_{2}Digital\;payments_{ijt}\\ \;+\gamma_{2}Control_{ijt}+\varphi_{i}+\delta_{t}+\varepsilon_{ijt} \end{array}$$

(4) $$\begin{array}{l} Expenditure_{ijt}=\alpha_{3}+\beta_{3}Digital\;payments_{ijt}+\theta Inter_{ijt}+\\ \;\gamma_{3}Control_{ijt}+\varphi_{i}+\delta_{t}+\varepsilon_{ijt} \end{array}$$

The *Inter* are the mediating variables, and the *Control* is the control variable, which is consistent with the baseline regression. The core coefficient of the mediation model is β, if the coefficients in models (2) and (3) are significant, and pass the mediating effect test and are consistent with the theoretical hypothesis, then the mediating effect exists.

#### Household Use of Big Data-Based Digital Payments Eases Household Credit Constraints

We use the household's loan balance to determine credit availability and thus measure the household's credit constraint status. If the household has any debt, then credit availability is equal to 1. If the household has no debt, then its credit availability is equal to 0. In the CFPS questionnaire, the relevant questions related to credit availability are: “Does your family have any outstanding bank loans to buy or build or renovate your house?”, “Apart from your mortgage, does your family have any other outstanding bank loans at present?” “Do you have any outstanding loans from organizations or individuals other than banks, such as private credit institutions, relatives, friends, acquaintances, etc., for the purchase or construction or renovation of your home?” and “Pending loans from friends, relatives and private individuals” Does your household owe money to organizations or individuals other than banks (e.g., private credit institutions, relatives, friends, acquaintances, etc.) for reasons other than borrowing for the purchase or construction of a house, and has it not been repaid?” If the household head answers “yes” to any of the above questions, the household is certified as having a loan month and their credit availability is 1.

As we can see from column (2) in Table 7, the use of digital payments by households has a significant positive effect on the credit availability of households. We can also see by column (3) that the coefficient of the core explanatory variable becomes smaller when credit availability is added to the regression. Moreover, both the Sobel test and the Boostrap test show that the mediating effect is significant at the 1% confidence level, a phenomenon that confirms the existence of a partial mediating effect and suggests that the mediating effect explains 2.3% of the total effect.

**Table 7:** Mechanism analysis: credit constraints.

	**(1)**	**(2)**	**(3)**
**Dependent variable**	**Household**	**Credit**	**Household**
	**healthcare**	**Availability**	**healthcare**
	**expenditure**		**expenditure**
Digital payments	0.0534**	0.00892***	0.0506*
	(2.07)	(2.86)	(1.95)
Credit Availability			0.315***
			(5.87)
Control variables for household head	Yes	Yes	Yes
Control variables for household	Yes	Yes	Yes
Household fixed effects	Yes	Yes	Yes
Time fixed effects	Yes	Yes	Yes
*N*	24,126	24,126	24,126
*R* ^2^	0.016	0.006	0.018
Sobel Z	2.803
Sobel Z-P values	(0.0005)
Bootstrap Z	2.60
Bootstrap Z-P values	(0.009)
Intermediary effect as a percentage	2.30%

^***^, ^**^, and ^*^ indicate significant at the 1, 5, and 10% statistical levels, respectively, with t-values in parentheses.

The reason is that households generate big data when they use digital payments, and these big data can empower both the supply side and demand measurement of financial services, reducing households' credit constraints and ultimately benefiting the increase of households' healthcare spending. Specifically, on the one hand, financial institutions use big data to analyze the payment behavior activities of households, which helps financial institutions accurately assess the risk status of households, thus the risk of financial institutions providing credit to households is reduced, and financial institutions have more incentives to provide loans to households. On the other hand, big data can provide households with precise information of various types, including not only information about credit services that are suitable for them, but also information about the household's financial income and employment. As a result, households are more likely to increase their applications for credit services, and their incomes are likely to increase. And higher-income households are more likely to have their credit applications approved. In summary, household use of big data-based digital payments promotes household healthcare spending by easing household credit constraints.

#### Household Use of Big Data-Based Digital Payments Increases the Social Capital of Households

We use household spending on human gifts to measure the social capital of the household. With respect to favor gift expenditure, the CFPS questionnaire addresses the question, “Including in-kind and cash, how many total favor gifts did your household give in the past 12 months?” In order to correct for bias, we talk about the expenditure on favors to be logarithmic. The specific regression results can be seen in Table 8. We can see through column (2) that the use of digital payments by households has a significant positive effect on social capital. We can also see through column (3) that the coefficients of the core explanatory variables become smaller when social capital is added to the regression. In addition, both the Sobel test and the Boostrap test show that the mediation effect is significant at the 1% confidence level, a phenomenon that confirms the existence of a partial mediation effect and suggests that the mediation effect explains 7.24% of the total effect.

**Table 8:** Mechanism analysis: social capital.

	**(1)**	**(2)**	**(3)**
**Dependent variable**	**Household**	**Social**	**Household**
	**healthcare**	**Capital**	**healthcare**
	**expenditure**		**expenditure**
Digital payments	0.0525*	0.0344*	0.0493*
	(1.93)	(1.86)	(1.84)
Social Capital			0.0951***
			(8.02)
Control variables for household head	Yes	Yes	Yes
Control variables for household	Yes	Yes	Yes
Household fixed effects	Yes	Yes	Yes
Time fixed effects	Yes	Yes	Yes
N	24,126	24,126	24,126
R2	0.016	0.046	0.025
Sobel Z	5.543
Sobel Z-P values	(0.000)
Bootstrap Z	5.880
Bootstrap Z-P values	(0.000)
Intermediary effect as a percentage	7.24%

^***^ and ^*^ indicate significant at the 1 and 10% statistical levels, respectively, with t-values in parentheses.

In other words, when households use digital payments based on big data, digital platforms exert social attributes. The digital platform uses big data to mine families' usage data and promotes subjects on the digital platform to families, such as relatives, friends, and medical institutions, in order to strengthen their dependency on the digital platform while also increasing their social capital. On the one hand, increasing a family's social capital allows them to share knowledge with other people and expand their information channels. Families may be able to receive more healthcare information via their social networks, lowering the cost of looking for healthcare information and allowing households to increase healthcare spending. On the other hand, increasing households' social capital allows them to be more closely connected with medical institutions, banks, and other subjects, allowing medical institutions and banks to better understand households' situations and provide health care services to them, thereby increasing household health care expenditures.

## Further Discussion: the Heterogeneity of Household Healthcare Expenditures

The previous analysis found that households' digital payment use increases households' healthcare spending by alleviating credit and information constraints. So, does this effect differ for households with different endowments? We examine the heterogeneous impact of digital payment use on households' health care expenditures separately from differences in financial availability, age of the household head, and education level of the household head.

### Financial Accessibility of Households

In this paper, we refer to Beck et al. (27) and use the number of bank branches per capita in each province as a proxy variable for traditional financial accessibility. After that, this paper divides the sample into two groups, low traditional financial accessibility and high traditional financial accessibility, based on the average of the number of bank branches per capita in each province. As seen in Table 9, the coefficient of digital payment in areas with low traditional financial accessibility is 0.0809 and is significant at the 5% level. This indicates that in places with high traditional financial accessibility, households can more easily participate in traditional financial services and have higher financial literacy. Therefore, this group can use digital payments more easily and give full play to the role of digital payments empowered by big data.

**Table 9:** Heterogeneity of household financial accessibility.

	**(1)**	**(2)**
	**Low financial**	**High financial**
	**accessibility**	**accessibility**
Digital payments	0.0294	0.0809^**^
	(0.89)	(2.29)
Control variables for household head	Yes	Yes
Control variables for household	Yes	Yes
Household fixed effects	Yes	Yes
Time fixed effects	Yes	Yes
*N*	14,092	10,034
*R* ^2^	0.017	0.017

** *Indicate significant at the 5% statistical levels, respectively, with t-values in parentheses*.

### The Age of the Household Head

The household head has a substantial impact on the household's decision-making, and age is an essential aspect of the household head. Based on whether the household head is <60 years old, we split the sample into a young and middle-aged group and an older group. Young and middle-aged household heads usually have a higher level of awareness, recognize the advantages of digital payments, and have a stronger willingness to use them. In addition, young and middle-aged heads of households usually have a higher risk tolerance. They are also more open-minded, and they are more willing to try new things. Most older heads of household may have a relatively lower level of awareness and are more traditional and conservative in their thinking. They will be more concerned about the risks associated with digital payments, such as suffering losses due to fraud, than the benefits that come with them. So they may be reluctant to use digital payments.

As seen in Table 10, in the young and middle-aged group, the coefficient of digital payments is 0.0563 and is significant at the 10% confidence level. This result is also more consistent with our common sense. Generally, younger household heads are more capable of learning, and they are receptive and willing to learn to use digital payments.

**Table 10:** Heterogeneity in the age of household heads.

	**(1)**	**(2)**
	**Young and middle-**	**Elderly (60 years**
	**aged people (18–59**	**old**
	**years old)**	**and above)**
Digital payments	0.0563^*^	0.0766
	(1.99)	(1.39)
Control variables for household head	Yes	Yes
Control variables for household	Yes	Yes
Household fixed effects	Yes	Yes
Time fixed effects	Yes	Yes
*N*	16,142	7,984
*R* ^2^	0.019	0.015

* *Indicate significant at the 10% statistical levels, respectively, with t-values in parentheses*.

### Education Level of the Household Head

We separated the sample into two groups, less educated and more educated, depending on whether the household head has fewer than 6 years of school, and then regressed them, because around 43.37 percent of household heads had <6 years of education. The results in Table 11 show that the coefficient of digital payments in the group with higher education level is 0.0536 and is significant at the 10% confidence level. These results suggest that the more educated household heads may have a stronger learning ability and are more receptive to new things such as digital payments, so they are able to take full advantage of digital payments and thus increase their healthcare spending.

**Table 11:** Heterogeneity in education level of household heads.

	**(1)**	**(2)**
	**Lower level**	**Higher level**
	**of education**	**of education**
Digital payments	−0.0454	0.0536^*^
	(-0.59)	(1.98)
Control variables for household head	Yes	Yes
Control variables for household	Yes	Yes
Household fixed effects	Yes	Yes
Time fixed effects	Yes	Yes
*N*	6,050	18,076
*R* ^2^	0.009	0.020

* *Indicate significant at the 10% statistical levels, respectively, with t-values in parentheses*.

## Conclusion

This article investigates the effect of big data-based digital payments on household healthcare expenditure from a big-data perspective. This research analyzes 24,126 sample data from 2014 to 2018 from the China Family Panel Studies (CFPS) to investigate the effect of big data-based digital payments on household healthcare expenditure. This research uses benchmark regression to examine the intermediary effect and heterogeneous influence of household digital payment on household health care expenditure, in order to improve knowledge of household health care expenditure and assist families in making better use of digital payment and maximizing its role in promoting a healthy lifestyle.

The following are the findings of this study. First, big data-based digital payments can dramatically raise household healthcare expenditure; that is, the higher a family's mobile spending, the higher the household healthcare expenditure. Second, the mechanism analysis demonstrates that by reducing household credit limitations and collecting social capital, the household application of big data-based digital payments can enhance household health care expenditure. Third, in families headed by middle-aged and young individuals with high education and financial access, the use of digital payment has a larger role in promoting household healthcare expenditure. Fourth, the conclusions of this work are still robust when using the instrumental variable technique, substituting the indicators of household healthcare expenditure, replacing the indicators of digital payment, and shortening the tail of the variables.

This paper, however, may have the following flaws. First, this article only looks at the influence of digital payments on household healthcare expenditure, but it ignores the impact on individual health outcomes, which is a finding that needs to be explored more in the future. Second, because China's digital payment includes a variety of types, including mobile payment, bank electronic payment, digital RMB payment, etc., this paper examines the impact of digital payment in general from the perspective of big data, rather than providing a more detailed classified discussion on digital payment. As a result, researching the effects of various digital payment methods is an area that has to be addressed in the future.

## Data Availability Statement

The original contributions presented in the study are included in the article/supplementary material, further inquiries can be directed to the corresponding author/s.

## Author Contributions

CL and DL contributed to conception and design of the study. SH organized the database. SS performed the statistical analysis. YT wrote the first draft of the manuscript. CL, DL, and ZW wrote sections of the manuscript. All authors contributed to manuscript revision, read, and approved the submitted version.

## Conflict of Interest

The authors declare that the research was conducted in the absence of any commercial or financial relationships that could be construed as a potential conflict of interest.

## Publisher's Note

All claims expressed in this article are solely those of the authors and do not necessarily represent those of their affiliated organizations, or those of the publisher, the editors and the reviewers. Any product that may be evaluated in this article, or claim that may be made by its manufacturer, is not guaranteed or endorsed by the publisher.
